# Molten Globule of Hemoglobin Proceeds into Aggregates and Advanced Glycated End Products

**DOI:** 10.1371/journal.pone.0072075

**Published:** 2013-08-26

**Authors:** Afshin Iram, Tauqeer Alam, Javed M. Khan, Taqi A. Khan, Rizwan H. Khan, Aabgeena Naeem

**Affiliations:** 1 Department of Biochemistry, Faculty of Life Sciences, Aligarh Muslim University, Aligarh, India; 2 Interdisciplinary Biotechnology Unit, Aligarh Muslim University, Aligarh, India; Oak Ridge National Laboratory, United States of America

## Abstract

Conformational alterations of bovine hemoglobin (Hb) upon sequential addition of glyoxal over a range of 0–90% v/v were investigated. At 20% v/v glyoxal, molten globule (MG) state of Hb was observed by altered tryptophan fluorescence, high ANS binding, existence of intact heme, native-like secondary structure as depicted by far-UV circular dichroism (CD) and ATR-FTIR spectra as well as loss in tertiary structure as confirmed by near-UV CD spectra. In addition, size exclusion chromatography analysis depicted that MG state at 20% v/v glyoxal corresponded to expanded pre-dissociated dimers. Aggregates of Hb were detected at 70% v/v glyoxal. These aggregates of Hb had altered tryptophan environment, low ANS binding, exposed heme, increased β-sheet secondary structure, loss in tertiary structure, enhanced thioflavin T (ThT) fluorescence and red shifted Congo Red (CR) absorbance. On incubating Hb with 30% v/v glyoxal for 0–20 days, advanced glycation end products (AGEs) were detected on day 20. These AGEs were characterised by enhanced tryptophan fluorescence at 450 nm, exposure of heme, increase in intermolecular β-sheets, enhanced ThT fluorescence and red shift in CR absorbance. Comet assay revealed aggregates and AGEs to be genotoxic in nature. Scanning electron microscopy confirmed the amorphous structure of aggregates and branched fibrils of AGEs. The transformation of α-helix to β-sheet usually alters the normal protein to amyloidogenic resulting in a variety of protein conformational disorders such as diabetes, prion and Huntington's.

## Introduction

Protein must acquire specific three-dimensional structure in order to achieve their biological functions [1]. There are various local and non-local interactions such as intra molecular hydrogen bonds, hydrophobic interactions etc. that stabilize the compact tertiary structure of proteins. Several studies have shown that aggregation can be induced in vitro by conditions that favour partially folded; Molten Globule (MG) -like states [2]. The accumulation of protein aggregates also termed as amyloids is observed in a number of neurodegenerative diseases like Alzheimer's, Parkinson's, prion diseases etc. [3]–[4].

Aggregation is promoted by post-translational modifications, such as chemical modifications at amino acid residues, including glycation, glycosylation, phosphorylation, sumoylation etc. One of these modifications such as glycation is due to incubation of protein with sugars/sugar derivatives like glyoxal, methylglyoxal and 3-deoxyglucosone for prolonged time period [5]. Protein glycation is a composite series of condensations, rearrangements, fragmentations, and oxidative modifications resulting in generation of Advanced Glycated End products (AGEs) [6] which further cause various pathologies [7]. These products possess a substantial amount of structural variations in the secondary and tertiary levels and consequently alter the functional properties of the proteins [8]. Glyoxal binds to free amino groups of proteins resulting in the formation of cross-linked aggregates [9]. High level of glyoxal in blood is associated with hyperglycemia condition in diabetes mellitus [10].

Hemoglobin (Hb), in its native structure, is a tightly folded tetrameric globular protein composed of the non-covalent association of heme-containing subunits (αβ)_2_ [11]. (αβ)_2_ tetramer dissociates reversibly into αβ dimer via breaking of bonds at the α_1_β_2_ and α_2_β_1_ interface. Despite of highly heterogeneous character of Hb, its dimers and the tetramers have very similar spectral and ligand-binding properties. Hb is chosen as a model protein for the study of conformational alterations as well as α to β transitions in the presence of glyoxal. Under these experimental conditions, both dimers and tetramers exist in rapid equilibrium. It contains heme prosthetic group with a molecular weight of 64,500 KDa. The α-helix is the most commonly encountered secondary structure of Hb molecule. Most of the amino acid residues in Hb are arranged in α-helices form.

Dicarbonyl induced structural alterations in proteins are important topic of research due to their role in forming AGEs. These AGEs have a role in the progression of diabetes [12]. As glyoxal and its derivatives are found in beer, wine, tea as well as commonly noticed in fermented food and beverages, hence emphasis should be given to lessen the harmful consequences of protein glycation.

The present work clearly demonstrates the formation of Hb aggregates and AGEs in the presence of different experimental conditions. During the aggregation pathway, MG state of Hb (at 20% v/v glyoxal) was observed. This MG state aggregates at 70% glyoxal on short term incubation (4 hrs) and forms AGEs at 30% glyoxal on long term incubation (20 days). By using non-physiological concentration of glyoxal in our study, a chemically equivalent glycation reaction similar to in vivo was mimicked. 40% aqueous solution of glyoxal is used as a preservative in nail polishes and enamels. We are using glyoxal concentration in the range of 0–90% in our studies to evaluate structural changes in Hb above these concentrations.

## Materials and Methods

### Materials

Bovine Hb (catalogue no. H2500), fluorescent probes, viz., ANS, CR as well as ThT were purchased from Sigma (St. Louis, U.S.A). Glyoxal, sodium azide, sodium phosphate monobasic, sodium phosphate dibasic were purchased from SRL (Mumbai, India). Sodium Phosphate mono and dibasic (pH 7) were used for buffer preparations.

Hb was dissolved in 20 mM phosphate buffer of pH 7 to prepare the stock solution of 80 µM and then dialyzed in the same buffer to remove impurities. Protein concentration was determined using molar extinction coefficient at 405 nm of 179 mM^−1^ per heme (Hb) on Hitachi single beam spectrophotometer using 1 cm path length [13].

### Effect of glyoxal on Hb

Samples of Hb in glyoxal were prepared separately with varying concentration i.e. 0–90% in 20 mM phosphate buffer, pH 7 and then incubated for 4 hr at 37°C before performing spectroscopic measurements. All the measurements were carried out at room temperature. Three replicates for each set were analyzed for the results. The final concentration of Hb in the incubation mixture at the start of the reaction was 3 µM.

### Preparation of glycated samples

For glycation studies, Hb was incubated in 30% glyoxal at 37°C for 20 days. The stock solution of 300 µM for Hb was prepared. The final concentration of Hb in the reaction mixture was 8 µM. All solutions were prepared under sterile condition by adding sodium azide (0.002%) to avoid contamination (bacterial or fungal). Aliquots were withdrawn on alternate day from the incubated samples up to 20 days for further studies.

### Intrinsic fluorescence measurements

The fluorescence spectra were recorded on a Shimadzu RF-5301 spectrofluorophotometer (Tokyo, Japan) using 1 cm path length quartz cell. The excitation wavelength was 280 nm and the emission was recorded in the range of 300–400 nm [14]. Final concentration of Hb was 3 µM.

### Acrylamide quenching

In the acrylamide-quenching experiments, aliquots of 5 M acrylamide stock solution were mixed with a protein stock solution (3 µM) to attain the required acrylamide concentration. Excitation was set at 295 nm in order to excite tryptophan fluorescence only, and the emission spectrum was monitored in the range of 300–400 nm. The slit width was set at 10 nm for both excitation and emission. The decrease in fluorescence intensity at λmax was analyzed according to the Stern-Volmer equation [15]:
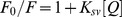
where F_0_ and F are the fluorescence intensities at an appropriate wavelength in the absence and presence of a quencher (acrylamide), respectively, K_sv_ is the Stern-Volmer constant for the collisional quenching process, and [Q] is the concentration of the quencher.

### Determination of free amino groups in glycated Hb

The free amino groups present in Hb at 30% and 40% glyoxal were detected by trinitrobenzene sulphonate (TNBS) method [16]. The absorbance was measured at 420 nm against a blank. The final concentration of Hb was 8 µM.

### 8-Anilino-1-Naphthalene-Sulphonic acid (ANS) fluorescence measurements

ANS binding was measured by fluorescence emission spectra with excitation at 380 nm and emission was recorded from 400 to 600 nm [17]. Typically, ANS concentration was 100 molar excess of the protein concentration and protein concentration was in the vicinity of 3 µM.

### Soret absorbance spectroscopy

Soret absorption of the heme group was monitored by a Shimadzu UV-1700 Spectrophotometer by using a 1 cm path length cell. The final concentration of protein in each sample was 3 µM and the reading was taken in the range of 350–700 nm [18].

### Circular Dichroism (CD) spectroscopy

CD spectra of protein samples were recorded on a J-810 Jasco CD spectropolarimeter calibrated with ammonium D-10-camphorsulfonate. Cells of path lengths 0.1 and 1 cm were used for scanning between 250–200 nm and 300–250 nm respectively. Each spectrum was the average of 4 scans [19]. The final concentration of protein in each sample was 8 µM for far-UV CD studies and 15 µM for near-UV CD studies. The results were expressed as the mean residue ellipticity (MRE in deg. cm^2^. dmol^−1^), which was defined as:
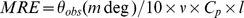
where *θ*_obs_, was the observed ellipticity in degrees (°), n the total number of residues, Cp the molar fraction, and ‘*l*’ the length of light path in cm.

### Attenuated *Total Reflection* Fourier Transform Infra Red (ATR-FTIR) spectroscopy

FTIR spectra were recorded with Interspec 2020 FTIR spectrometer in deuterated water at room temperature. Sample aliquots were placed between CaF_2_ windows separated by a 50 µm polyethylene terephthalate spacer. The sample compartment was thoroughly purged with dry nitrogen. Protein concentration was 80 μM. The scanning wavenumber was from 1000 to 4000 cm^−1^ [20].

### Size exclusion chromatography (SEC) experiment

SEC experiments were carried out on a Sephadex G 200 (76×1.15 cm) column. The column was pre-equilibrated with 20 mM phosphate buffer of pH 7 and in the presence of 20% and 70% glyoxal. 2 ml of 30 μM native and Hb in the presence of 20% and 70% glyoxal were loaded to the column and eluted at 20 ml/h. The eluted fractions were read at 280 nm. The molecular weight marker used were urease (590,000), myosin (500,000) glucose oxidase [GOD] (160,000), Concanavalin A [con A] (104,000), bovine serum albumin [BSA] (66,700), Hb (64,500), α1 antitrypsin (52,000), ovalbumin (45,000) and chymotrypsinogen [Chy] (25,500).

### Dynamic Light Scattering (DLS) measurements

DLS measurements were carried out at 830 nm by using DynaPro-TC-04 dynamic light scattering equipment (Protein Solutions, Wyatt Technology, Santa Barbara, CA) equipped with a temperature-controlled micro sampler. Hb (30 μM) with glyoxal was incubated for 4 hrs. The solutions were spun at 10,000 rpm for 10 min and were filtered serially through 0.22 and 0.02 mm Whatman syringe filters directly into a 12 ml quartz cuvette. For each experiment, 20 measurements were taken. Mean hydrodynamic radius (R_h_) and polydispersity were analyzed using Dynamics 6.10.0.10 software at optimized resolution. The R_h_ was estimated on the basis of an auto correlation analysis of scattered light intensity data based on translation diffusion coefficient by Stoke's-Einstein relationship-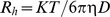
where R_h_ is the hydrodynamic radius, k is Boltzmann constant, T is temperature, η is the viscosity of water and D is diffusion coefficient [21].

### Thioflavin T (ThT) fluorescence assay

ThT fluorescence was measured in a 1 cm path length quartz cell to monitor aggregation of Hb. The following parameters were adjusted for monitoring ThT fluorescence intensity during aggregation experiments: λ_ex_ = 440 nm, λ_em_ = 460–600 nm [22]. Final concentration of protein in the sample was 3 µM whereas the concentration of ThT was 15 µM.

### Congo Red (CR) assay

The formation of aggregates was probed by Congo Red absorbtion spectra recorded in 400 to 700 nm range. For this experiment, 240 µl (6 µM) aliquots of the protein solutions were withdrawn and mixed with 260 µl of a solution containing 20 µM Congo Red and 20 mM sodium phosphate buffer, pH 7 [23].

### Comet assay of Hb aggregates

Isolated lymphocytes were exposed to aggregates of Hb (after removing glyoxal by air drying) in a total reaction volume of 1.0 ml of 20 mM phosphate buffer pH 7.2. Incubation was performed at 37°C for 1 hr. After incubation, the reaction mixture was centrifuged at 716.8 g, the supernatant was discarded and pelleted lymphocytes were resuspended in 100 μL of PBS and processed further for Comet assay. Comet assay of aggregated protein was performed under alkaline conditions [24].

### Scanning Electron Microscopy (SEM) studies

The micro-architecture of Hb aggregates was observed using scanning electron microscopy. Air dried samples were adsorbed onto cellulose ultrafiltration membrane then gold coated and imaged using a JSM-6510LV (JEOL JAPAN) scanning electron microscope running.

## Results

### Intrinsic fluorescence measurements

**Figure 1:**
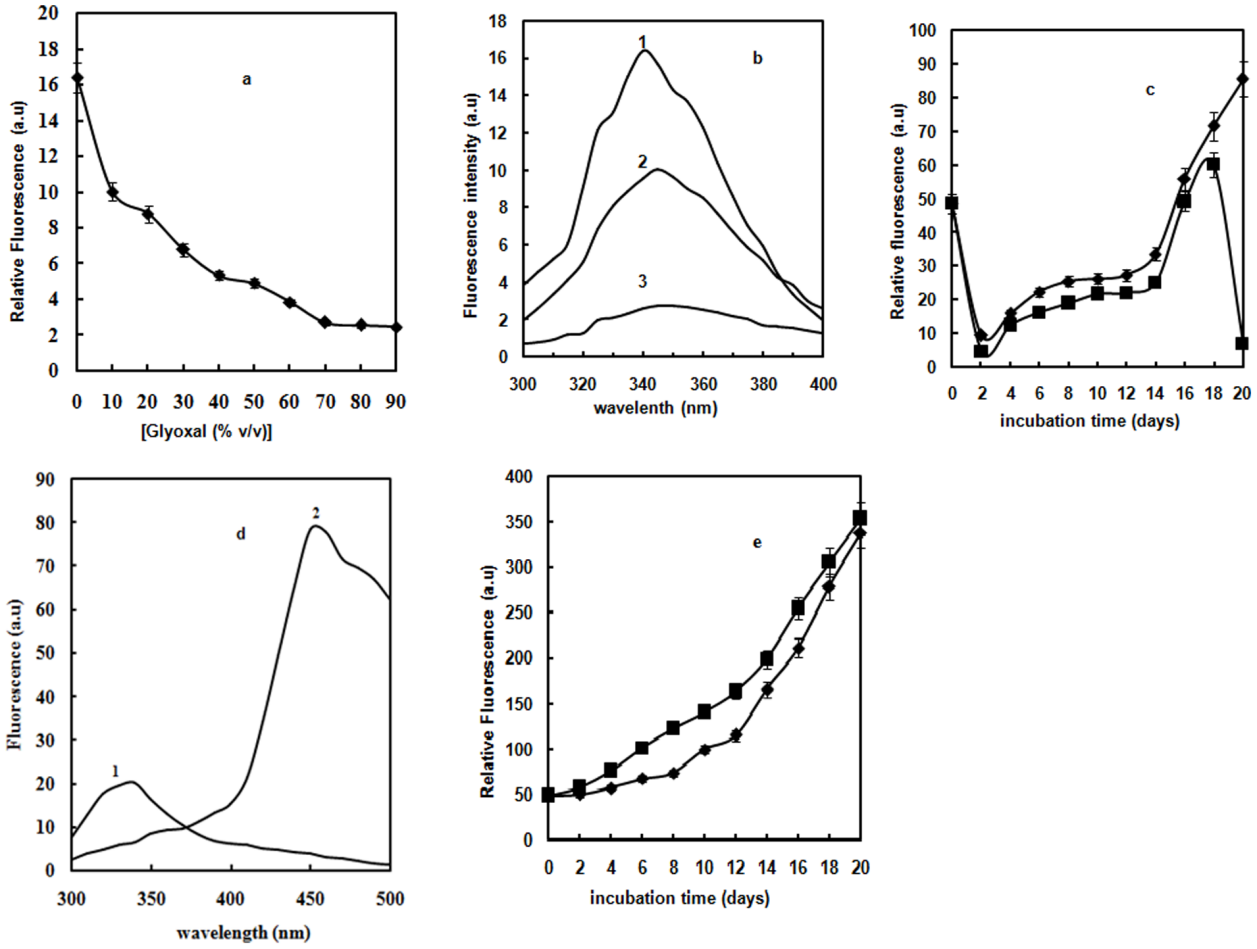
Fluorescence studies. (**a**) Relative fluorescence of Hb as a function of glyoxal [♦]. Error bars represent the mean ± SD (n = 3). *Significance p<0.05 with respect to control. (**b**) Intrinsic fluorescence emission spectra of Hb in the absence of glyoxal (curve 1); in the presence of 20% (curve 2) and 70% glyoxal (curve 3). (**c**) Relative fluorescence of Hb as a function of increasing days at 30% [♦] and 40% [▪] glyoxal excited at 280 nm at 37°C. The protein concentration was 8 µM and the path length was 1 cm. Error bars represent the mean ± SD (n = 3). *Significance p<0.05 with respect to control. (**d**) Intrinsic Fluorescence emission spectra of native Hb in 20 mM sodium phosphate buffer of pH 7 (curve 1) and Hb at 30% glyoxal on day 20 at 37°C (curve 2). The excitation wavelength was 280 nm and emission wavelength was in the range 300–500 nm. The protein concentration was 3 µM and the path length was 1 cm. (**e**) Time dependent formation of two AGEs products of Hb, excitation at 330 nm (▪) and 365 nm [♦] at 30% glyoxal at 37°C. The protein concentration was 8 µM and the path length was 1 cm. Error bars represent the mean ± SD (n = 3). *Significance p<0.05 with respect to control.

Intrinsic fluorescence is a sensitive index of alterations in protein conformation. Hb contains six Trp and ten Tyr residues which are restricted to hydrophobic regions closer to the heme group [25]. The contribution of glyoxal alone to the emission spectra was taken into account and here we reported the subtracted fluorescence spectra. Fluorescence properties of Hb molecules incubated with varying concentrations of glyoxal were observed through the maximum emission peak. There was regular decrease in emission maxima of Hb on increasing glyoxal concentration, reaching the plateau at 70% glyoxal (Fig. 1a). Native Hb possesses maximum fluorescence intensity around 340 nm due to much accessibility of Trp residues to the solvent (curve 1 of Fig. 1b) [26]. However, as the glyoxal concentration was increased to 20%, Trp fluorescence intensity significantly decreased with a red shift of 5 nm relative to native (curve 2). This is due to the transfer of Trp residues to a more polar environment [27]. At 70% glyoxal, there was further reduction in fluorescence intensity compared to 20% glyoxal (curve 3). 70% glyoxal causes denaturation of the protein resulting in exposure of Trp to water hence quenched Trp fluorescence was found. From our fluorescence data, we concluded the altered Trp milieu in Hb at 20% glyoxal and quenched Trp fluorescence at 70% glyoxal. 30% and 40% aqueous glyoxal give burning sensations to skin, depending on the application time in vivo studies. These results thus prompted us to further investigate structural alterations of Hb on incubating it at mid range of glyoxal concentration i.e. 30% and 40% for various days. Hence, glycation studies were performed to check the structural alterations in Hb. The relative Trp fluorescence plot of Hb in presence of 30% and 40% glyoxal as a function of increasing days indicate alterations in protein structure (Fig. 1c). Trp fluorescence of Hb on incubating up to 20 days in presence of 30% and 40% glyoxal, indicate that 30% glyoxal is more effective than 40% glyoxal in inducing glycation reaction. However, Hb aggregates at 40% concentration of glyoxal. On day 20, native Hb showed λ_max_ at 340 nm (curve 1 of Fig. 1d); in the presence of 30% glyoxal, reduction in fluorescence intensity was observed (curve 2). In addition to the decreased fluorescence intensity at 340 nm, a new peak emerged at 450 nm on day 20. The observed decrease in Trp fluorescence (at 340 nm) and simultaneous increase of emission from the glycation products (at 450 nm) can be explained by fluorescence resonance energy transfer (FRET) from Trp to the AGEs. The time dependent relative Trp fluorescence plot of glycated Hb in the presence of 30% glyoxal is depicted in Figure 1e. The temporal accumulation of possibly two AGE products i.e. pentosidine and malonaldehyde (excitation at 330 nm and 365 nm respectively) on day 20 were detected. The pentosidine type of AGE products seems to be rich in the presence of 30% glyoxal on day 20.

### Acrylamide quenching

**Figure 2:**
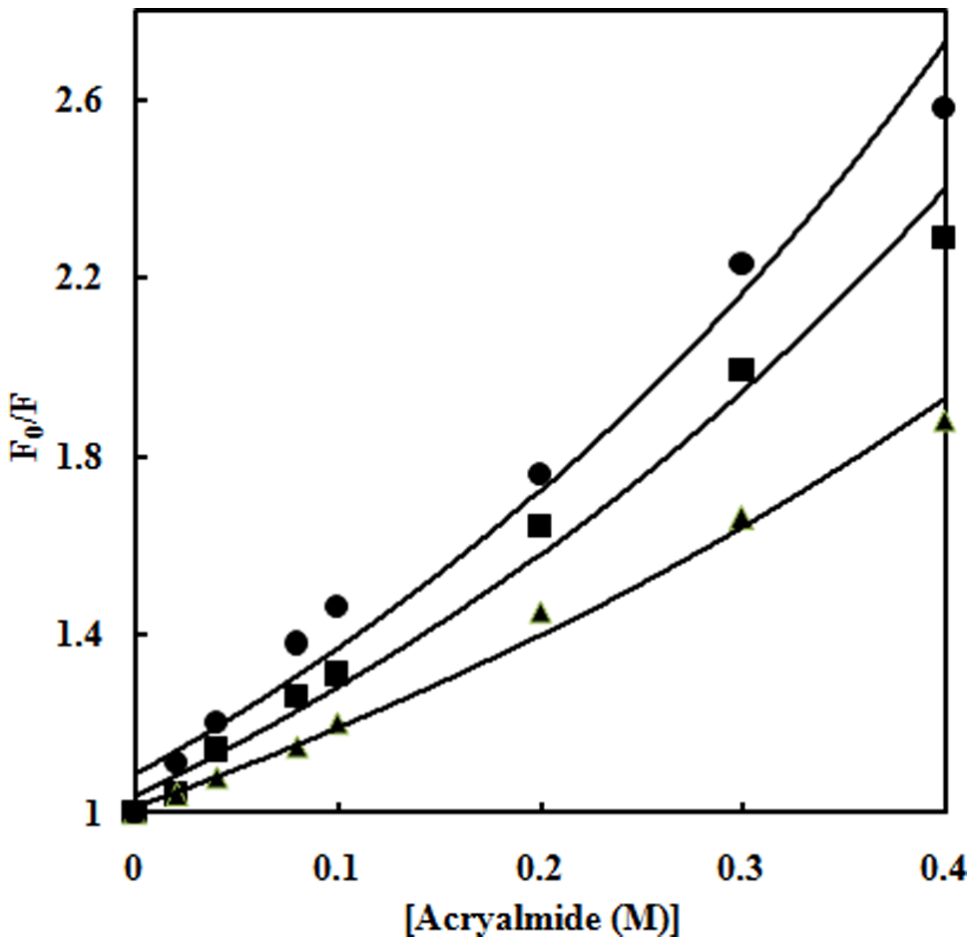
Acrylamide quenching. Stern–Volmer plots for acrylamide quenching of tryptophan fluorescence of native Hb in 20 mM sodium phosphate buffer, pH 7 (▴); Hb at 20% (▪) and 70% glyoxal [•]. Values shown are the ratios of fluorescence in the absence of acrylamide (F_0_) to the fluorescence at that concentration of quencher (F). The excitation wavelength was 295 nm and emission wavelength was in the range 300–400 nm. The protein concentration was 3 µM and the path length was 1 cm.

To rule out the possibility that the change in intrinsic fluorescence intensity upon increasing glyoxal concentration is only due to fluorescence quenching of Trp, acrylamide quenching experiment was performed for Hb at 0, 20% and 70% glyoxal (Fig. 2). The quenching data analysed from Stern-Volmer plots are shown in Table 1. Quenching by acrylamide resulted in linear Stern-Volmer plots in absence and presence of glyoxal. Trp residues in native Hb impart the least accessibility to acryalmide quencher. The K_SV_ values of Hb at 20% and 70% glyoxal were higher as compared to native Hb [28]. This indicates that Trp residues in molten globule and aggregates possess much accessibility to the quencher due to the greater distance between Trp residues and heme group.

**Table 1:** Acrylamide quenching parameters of Hb in different conditions.

Conditions	K_sv_ (M^−1^)
Native Hb	2.22
20% glyoxal treated Hb	3.26
70% glyoxal treated Hb	3.88

### Determination of free amino groups in glycated Hb

**Figure 3:**
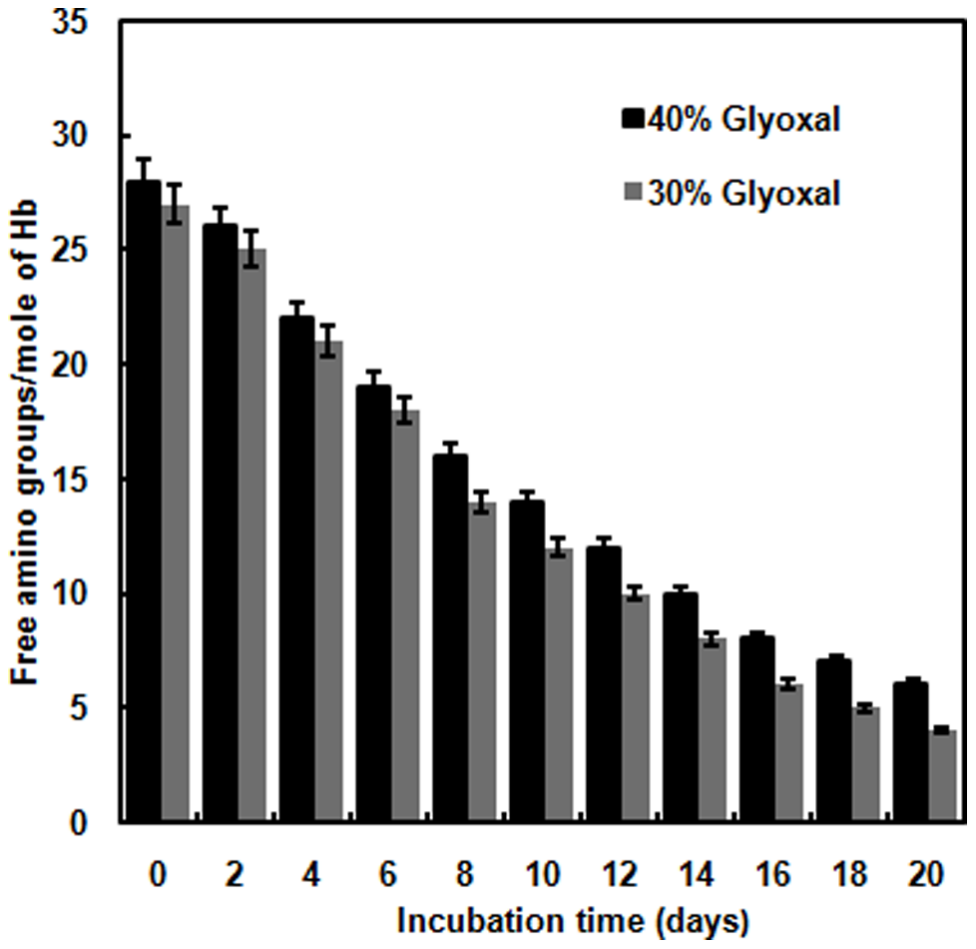
TNBS assay. Bar graph shows free amino groups present per mole of Hb incubates with 30% and 40% glyoxal as a function of increasing days. Error bars represent the mean ± SD (n = 3). *Significance p<0.05 with respect to control. The absorbance was recorded at 420 nm against a blank. The protein concentration was 8 µM and the path length was 1 cm.

In the beginning of the glycation reaction, free amino groups of protein such as ε-NH_2_ groups of lysine interact with the carbonyl groups resulting in loss of free amino groups. On incubating Hb with 30% and 40% glyoxal, simultaneous decrease in number of free amino groups up to 20 days was significantly observed (Fig. 3). Maximum loss was seen for 30% glyoxal on day 20 compared to that for 40% concentration [29].

### ANS fluorescence measurements

**Figure 4:**
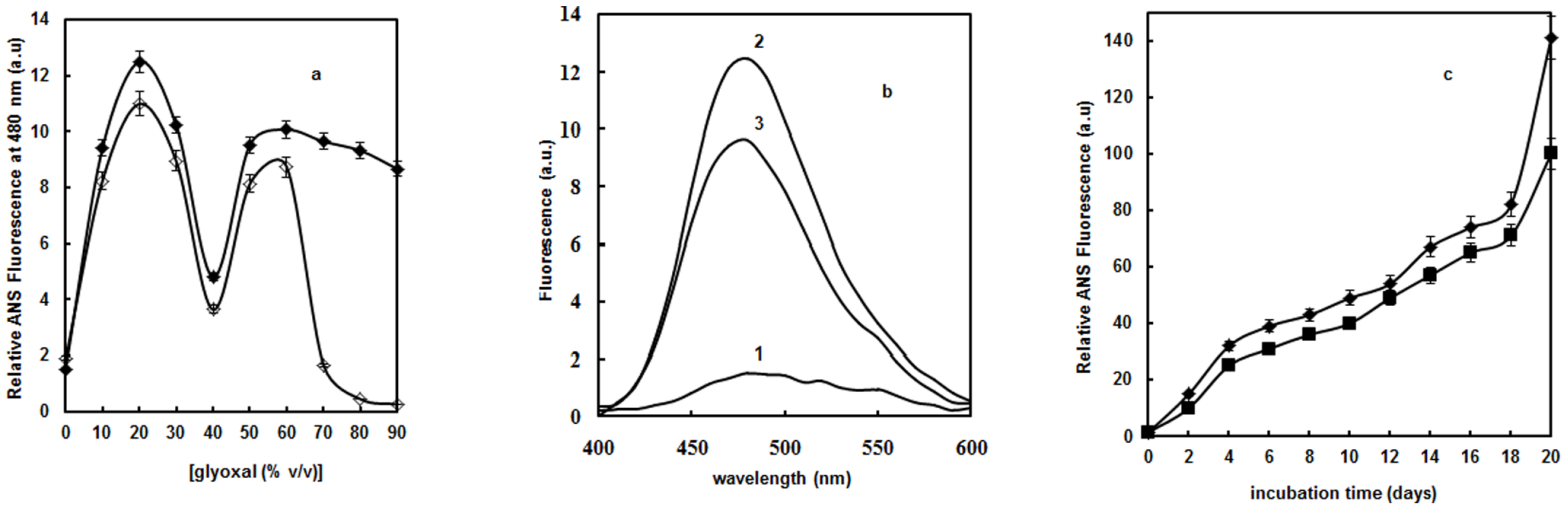
ANS Fluorescence studies. (**a**) Relative ANS fluorescence of Hb as a function of glyoxal before [⧫] and after centrifugation [◊]. Error bars represent the mean ± SD (n = 3). *Significance p<0.05 with respect to control (**b**) ANS fluorescence emission spectra of native Hb in absence of glyoxal (curve 1); in the presence of 20% glyoxal (curves 2) and 70% glyoxal (curve 3). The excitation wavelength was 380 nm and emission wavelength was in the range 400–600 nm. The protein concentration was 3 µM and the path length was 1 cm. (**c**) Relative ANS fluorescence of Hb as a function of increasing days at 30% [♦] and 40% [▪] glyoxal excited at 280 nm at 37°C. The protein concentration was 8 µM and the path length was 1 cm. Error bars represent the mean ± SD (n = 3). *Significance p<0.05 with respect to control.

ANS is an extensively utilized fluorescent probe for the characterization of MG states and aggregates. The emission spectrum of glyoxal alone was taken into account and here we reported the subtracted ANS fluorescence spectra. On varying glyoxal concentration from 0–90%, a notch is observed in Hb at 20% with prominent ANS intensity (Fig. 4a). It indicates that a MG state is appearing at 20% during the conformational transitions from native to glyoxal induced state. This state was followed by a reduction in ANS intensity up to 40% glyoxal concentration. Upon centrifugation, no significant changes in ANS fluorescence intensity were observed for Hb ranging from 0 to 60% glyoxal except a dramatic decrease in ANS fluorescence intensity at 70% glyoxal. Hence it can be concluded that Hb at 70% glyoxal formed aggregates. As seen by ANS fluorescence emission spectra (Fig. 4b), native Hb possesses negligible ANS fluorescence intensity (curve 1). Hb at 20% glyoxal (curve 2) possesses six fold enhanced ANS fluorescence intensity relative to native Hb (curve 1). The remarkable increase in fluorescence intensity and wavelength shift from 500 to 480 nm upon ANS addition to protein at 20% glyoxal is a clue that clusters of buried hydrophobic groups become accessible for ANS binding upon partial unfolding. In its native state these hydrophobic patches are buried and inaccessible for dye (curve 1). Decrease in ANS binding of Hb observed at 70% glyoxal (curve 3) is due to burial of hydrophobic patches that occurred owing to intermolecular protein-protein interactions. These results are consistent with Trp fluorescence studies where Hb at 20% glyoxal showed altered microenvironment. At 40% glyoxal, unfolded state was observed which upon further increase to 70% concentration led to aggregation. This partially unfolded state of Hb may disrupt its tertiary structure and form aggregates at 70% glyoxal due to intermolecular interactions among protein molecules.

As glyoxal is commercially supplied in the form of formalin (40% aqueous solution) to be used a cross-linking agent. This prompted us to further investigate the structural alterations; Hb was incubated with 30% and 40% glyoxal for 0–20 days (Fig. 4c). As seen from the figure, ANS related changes in fluorescence intensity in the presence of 30% glyoxal are more dominant relative to 40% glyoxal. This is attributable to the significant conformational alterations occurring in the Hb structure as a result of increase in hydrophobicity of the protein that triggers the formation of AGEs [8]. In contrast to this, there was initiation of aggregation at 40% glyoxal.

### Soret Absorption spectroscopy

**Figure 5:**
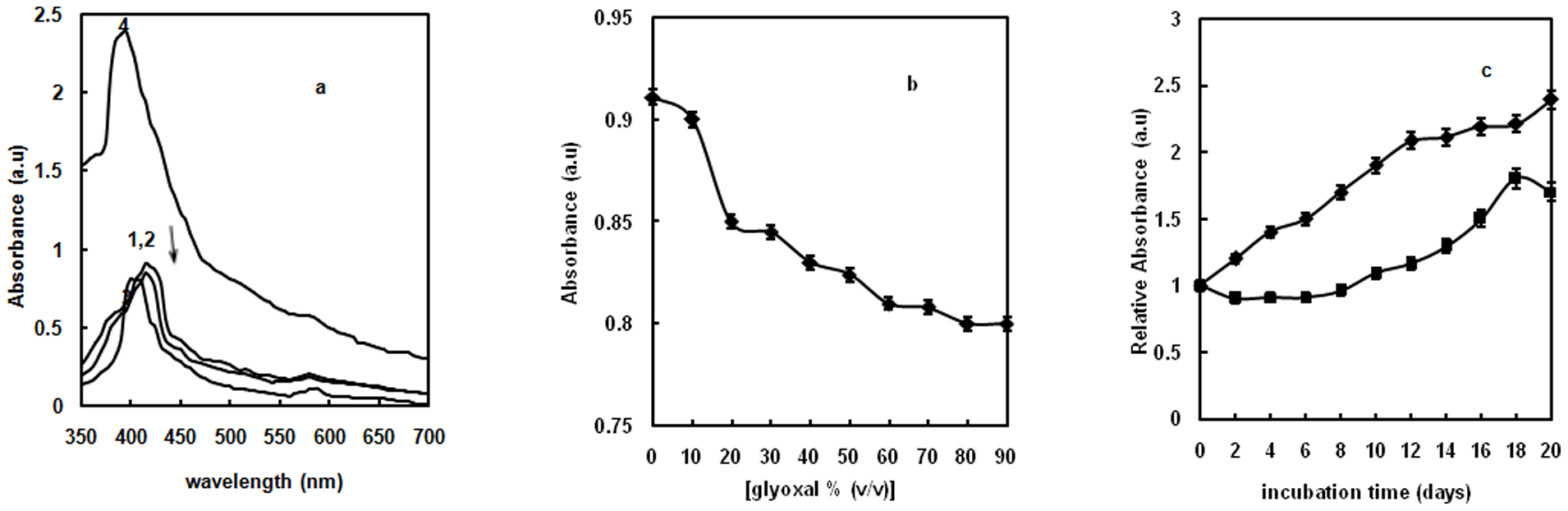
Soret spectroscopy. Soret Absorption spectra of native Hb in absence of glyoxal (curve 1); at 20% (curve 2); 70% (curve 3), and 30% glyoxal on day 20 (curve 4). The protein concentration was 3 µM and range was 350–700 nm. **(b)** Soret Absorbance of Hb at 410 nm in 20 mM sodium phosphate buffer, pH 7 as a function of increasing concentration of glyoxal. The protein concentration was 3 µM. Error bars represent the mean ± SD (n = 3). *Significance p<0.05 with respect to control. **(c)** Soret Absorbance of Hb at 410 nm in 20 mM sodium phosphate buffer, pH 7 as a function of increasing days in the presence of 30% [♦**]** and 40% [▪] glyoxal. The protein concentration was 8 µM. Error bars represent the mean ± SD (n = 3). *Significance p<0.05 with respect to control.

Hb displays characteristic absorption spectrum in the 350–700 nm range (Fig. 5a). The UV–vis spectrum of Hb solution shows a Soret band at 415 nm [30] and Q band at 580 nm in sodium phosphate buffer, pH 7 (curve 1). The locations of the Soret absorption band of iron heme may provide information about the denaturation of heme containing proteins. Absorbance of glyoxal alone is taken into account and here we showed the subtracted spectra. At 20% glyoxal, the absorbance was very close to that of native Hb solution (curve 2). This showed that Hb molecule retained its heme group at 20% glyoxal. At 70% glyoxal, the soret peak at 415 nm showed a blue shift of 10 nm i.e. peak at 405 nm (curve 3) with decreased absorbance. This suggests that heme is becoming more exposed to solvent than in the native structure because of denaturation of Hb. Relative plot of Soret region of Hb as a function of increasing glyoxal concentration is depicted in Figure 5b. On increasing glyoxal concentration, decrease in absorbance was observed reaching minimum absorbance at 70% glyoxal. This suggests that Hb became denatured at 70% glyoxal. Advanced studies are performed to monitor further alterations in heme group due to glycation. Therefore, Hb was incubated with 30% and 40% glyoxal for 0 to 20 days and their relative absorbance was observed as a function of increasing days (Fig. 5c). These results are also consistent with Trp and ANS fluorescence results suggesting that 30% glyoxal is the suitable concentration implied for glycation studies. Upon incubation with 30% glyoxal up to 20 days, Hb showed striking increase in Soret band with a blue shift of 20 nm relative to native probably due to formation of AGEs on day 20 (curve 4 of Fig. 5). It is possible that blue shift in Soret region occurred due to alteration of the steric pattern of Hb and hence affects the strength of heme- globin linkage [31]. These results suggest that the heme moiety is more exposed to the solvent probably due to disruption of non-covalent interactions of Hb molecule at 70% glyoxal, and 30% glyoxal on day 20 due to glycation. Alternatively, heme moiety remains intact at 20% glyoxal. It is also supported by this experiment that MG state of Hb is formed at 20% glyoxal that is prone to form aggregates at 70%, and AGEs at 30% glyoxal on day 20.

### Far-UV CD studies

**Figure 6:**
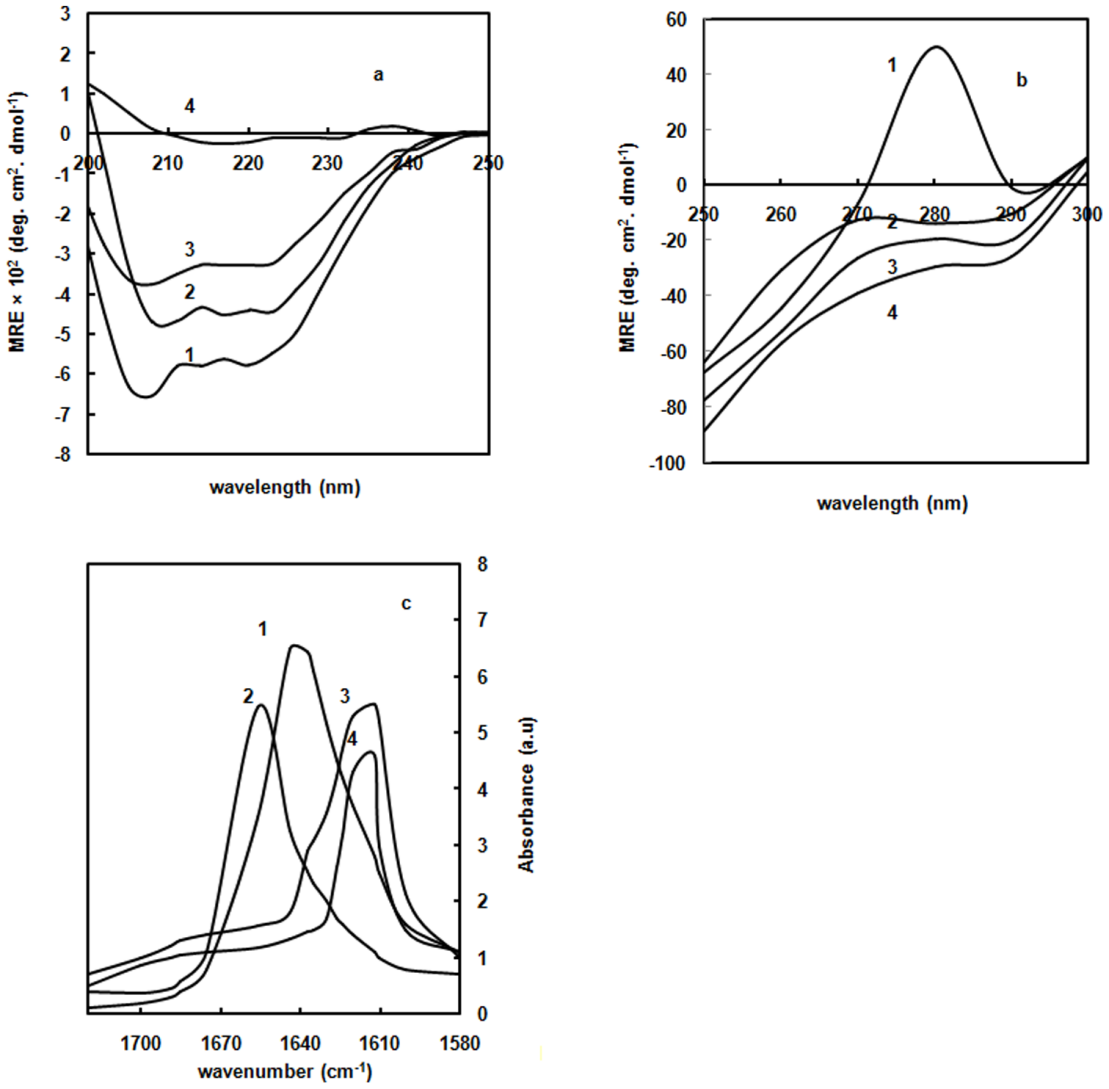
Secondary structure measurements. (**a**) Far-UV CD spectra of native Hb in absence of glyoxal (curve 1); in the presence of 20% (curve 2); 70% glyoxal (curve 3), and 30% glyoxal on day 20 (curve 4). The far-UV CD spectra were recorded between 200 nm and 250 nm. The protein concentration was 8 µM and the path length was 0.1 cm. (**b**) Near-UV CD spectra of native Hb in absence of glyoxal (curve 1); in the presence of 20% (curve 2); 70% (curve 3), and 30% glyoxal on day 20 (curve 4). The near-UV CD spectra were recorded between 300 and 350 nm. The protein concentration was 15 µM and the path length was 1 cm. (**c**) ATR-FTIR spectra of native Hb in absence of glyoxal (curve 1); in the presence of 20% glyoxal (curve 2); 70% glyoxal (curve 3), and 30% glyoxal on day 20 (curve 4). The protein concentration was 80 µM.

The far-UV CD spectra of proteins are highly sensitive towards protein structure, especially useful for determining the changes in protein secondary structure. Far-UV CD spectra of glyoxal alone was also monitored and here subtracted spectra was reported. Native Hb showed minima at 208 nm and 222 nm as indicative of α-helical structure (curve 1 of Fig. 6a). Addition of 20% glyoxal slightly influenced the CD spectra in respect of decreasing the MRE values at 208 nm and 222 nm (curve 2). This suggests that retention of helicity is observed in the presence of 20% glyoxal confirming the presence of MG state. Addition of 70% glyoxal resulted in the loss of the MRE value at 208 nm and shift of 222 nm peak to 220 nm (curve 3). Furthermore, upon incubating Hb with 30% glyoxal for 20 days (curve 4), peak was shifted from 220 nm to 217 nm retaining only β-sheets on day 20. These results indicate that Hb forms intermolecular β-sheets possibly due to aggregates formed at 70% glyoxal. The cross-linked intermolecular β-sheets are also appeared in presence of glycated Hb (AGEs) at 30% glyoxal on day 20.

### Near-UV CD studies

Near-UV CD spectra of proteins reflect contribution of aromatic side chains and disulfide bonds. The near-UV CD shoulder at 280–290 nm is allocated to the aromatic residues. These residues impart Hb a characteristic shoulder in the near-UV CD spectra around 280 nm (curve 1) as shown in Figure 6b. This suggests that native Hb has a well defined tertiary structure. A near-UV CD spectrum of glyoxal alone was also monitored and here subtracted spectra are reported. Addition of 20% glyoxal to Hb resulted in diminished shoulder at 280 nm to a negative value signifying the loss of native tertiary structure (curve 2). These results may coincide with a definition of the MG state: a “compact globule with native-like secondary structure and no prominent tertiary structure”. Therefore, Hb in presence of 20% glyoxal can be considered as MG state. In fact, this finding is observed for most proteins during their acid-induced unfolding stages [32]. Loss in tertiary structure in the MG state results in some internal nonpolar groups becoming exposed to the solvent, thus making the surface of the protein more hydrophobic compared to native state. With further increase in glyoxal concentration up to 70%, decrease in MRE relative to Hb at 20% glyoxal was observed (curve 3). On incubating Hb with 30% glyoxal for 20 days (curve 4), further loss in MRE value with pronounced red shift than to Hb at 70% glyoxal is detected on day 20. It is assumed that Tyr and Trp residues in the glyoxal incubated solutions may get buried due to intermolecular protein-protein interactions between Hb molecules.

### ATR-FTIR spectroscopy

ATR-FTIR spectroscopy has been used to examine the conformationally sensitive amide I' bands of Hb. Native Hb showed a peak in the amide I' region at 1644 cm^−1^ suggesting the presence of α-helical structure (curve 1 of Fig. 6c) [33]. At 20% glyoxal (curve 2), the peak was blue shifted to 1656 cm^−1^ which was significantly lower in intensity compared to native. It retained the native-like α-helical structure. At 70% glyoxal (curve 3), peaks at 1612 cm^−1^ and 1685 cm^−1^ with significantly lower intensity was observed as symptomatic of intermolecular β-sheet conformations. Similarly, on incubating Hb with 30% glyoxal for 20 days (curve 4), peak at 1612 cm^−1^ and 1685 cm^−1^ were observed with further decrease in intensity. These data correspond to the CD results signifying that secondary structures of native Hb is retained up to 20% glyoxal. This indicates that Hb forms MG state at 20% glyoxal. Further increasing the concentration of glyoxal up to 70%, increase in protein-protein interactions occurred owing to the formation of intermolecular β-sheets. Upon incubating Hb with 30% glyoxal for 20 days, cross-linking occurred due to formation of AGEs on day 20 which in turn increase the intermolecular β-sheets.

### SEC

**Figure 7:**
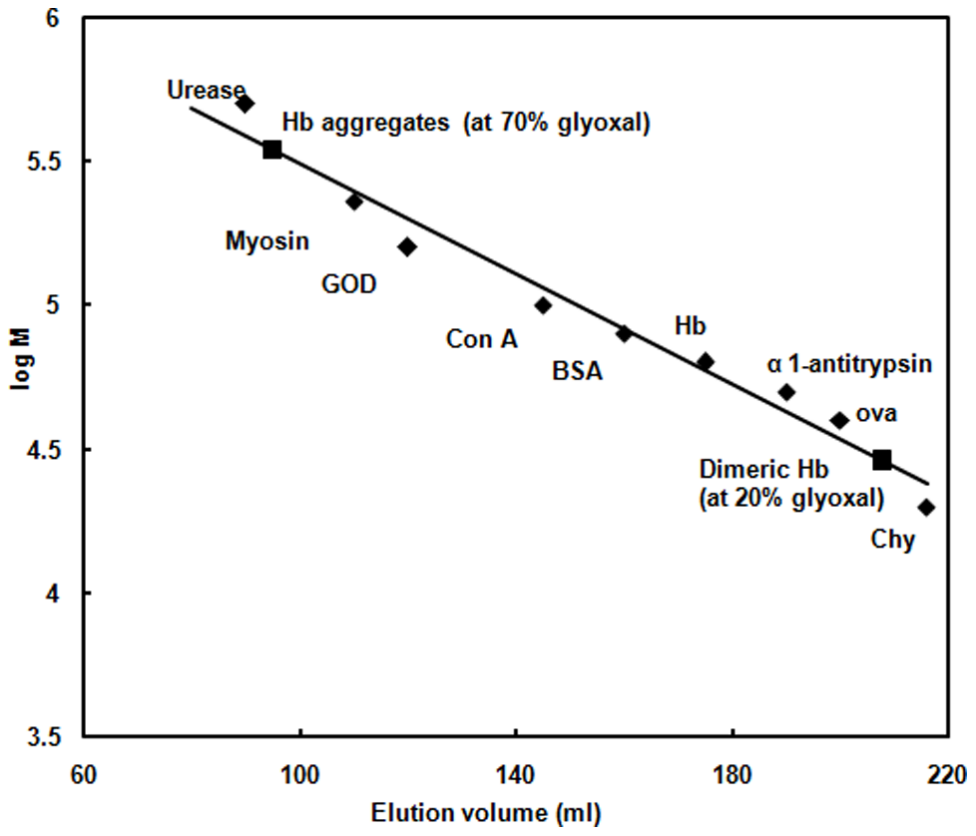
SEC. Gel filtration on Sephadex G-200 of native and Hb at 20% and 70% glyoxal [▪]. The molecular weight marker such as urease, myosin, glucose oxidase [GOD], Concanavalin A [con A], bovine serum albumin [BSA], Hb, α1 antitrypsin, ovalbumin, chymotrypsin (Chy) were labelled by [♦**]**. The protein concentration was 30 µM. The tetrameric and dimeric forms of Hb were eluted in phosphate buffer of pH 7.

Hb in absence and presence of glyoxal was subjected to SEC on a Sephadex G-200 column to study about tetramer-dimer configuration. The elution volume of one of the eluted fractions was equivalent to the molecular mass of native tetrameric Hb. The column was also calibrated with molecular markers such as urease, myosin, GOD, Con A, BSA, Hb, α1-antityrpsin, ovalbumin and Chy. Addition of 20% glyoxal showed an increased elution volume corresponding to a dimeric Hb as compared to native (Fig. 7). This suggests that the dimensions of the molecule have been decreased in presence of 20% glyoxal leading to the formation of MG state due to disruption of tertiary contacts as supported by CD and FTIR results. However, at 70% glyoxal, decrease in the elution volume corresponds to formation of Hb aggregates as compared to native due to formation of intermolecular interactions between Hb moieties. This indicates that the dimensions of the molecule have been increased in size in presence of 70% glyoxal.

### DLS measurements

**Figure 8:**
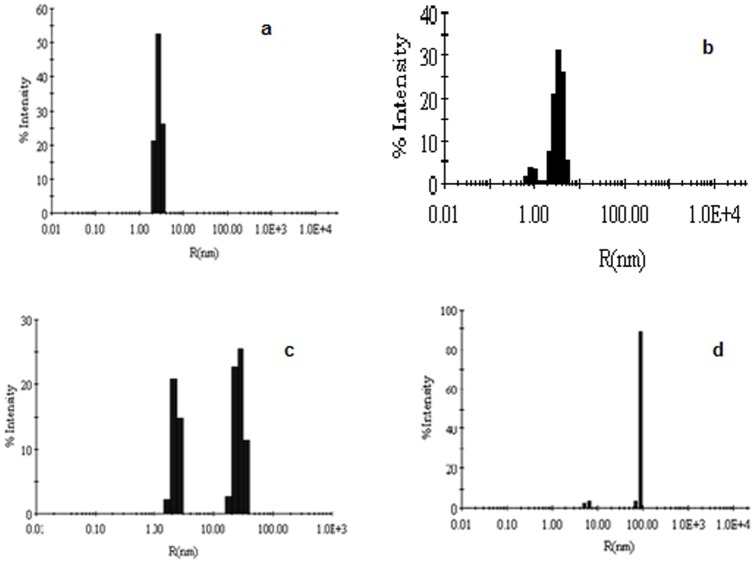
DLS results. Determination of hydrodynamic radii (R_h_) of (**a**) native Hb in absence of glyoxal (**b**) in presence of 20%; (**c**) 70% and (**d**) 30% glyoxal on day 20. The protein concentration was 30 µM.

DLS was employed to determine hydrodynamic radii (R_h_) of native Hb in absence and presence of glyoxal. In Figure 8, R_h_ of native Hb, Hb in presence of 20%, 70% glyoxal and 30% glyoxal on day 20 were calculated as in Table 2. The lower values of polydispersity (16.3) are indicative of homogenous species in the solution. The R_h_ value of 2.8 nm for native Hb is consistent with the previous findings (Fig. 8a) [34]. However, there were two different polydispersity values observed in Hb at 20% glyoxal suggesting that Hb forms heterogenous species in the presence of this concentration (Fig. 8b). The R_h_ of Hb in presence of 20% glyoxal was smaller in size in contrast to native. As seen from the data, some of the subunits of tetrameric Hb at 20% glyoxal dissociated into dimeric forms with an expanded hydrodynamic radius to form MG state in contrary to native.

**Table 2:** DLS data of Hb at different concentrations and conditions of glyoxal.

Conditions	R_h_ (nm)	Polydispersity (%)	App. MWt (kDa)
Native Hb	2.8	16.3	60.00
Hb +20% glyoxal	1.0, 2.9	11.5, 18.2	30, 62
70% glyoxal treated Hb	2.9, 28.4	13.7, 29.2	65, 546
30% glyoxal treated Hb at day 20	5.9, 87.6	11.2, 44.4	215, 118028

Two different polydispersity values in Hb were recorded at 70% (Fig. 8c) and 30% glyoxal (Fig. 8d) suggesting the existence of heterogenous species in these respective solutions. However, the R_h_ of Hb in presence of 30% glyoxal on day 20 was higher relative to Hb at 70% glyoxal suggesting that there are quiet larger aggregates observed here. The increase in R_h_ values on addition of glyoxal might be due to the elongation of protein shape around glyoxal [35]. This response might result in an increase in molecular volume due to aggregation. These light scattering data along with apparent molecular weight supported our presumption that protein at 70% glyoxal and 30% glyoxal (on day 20) forms small and large aggregates.

### ThT assay

**Figure 9:**
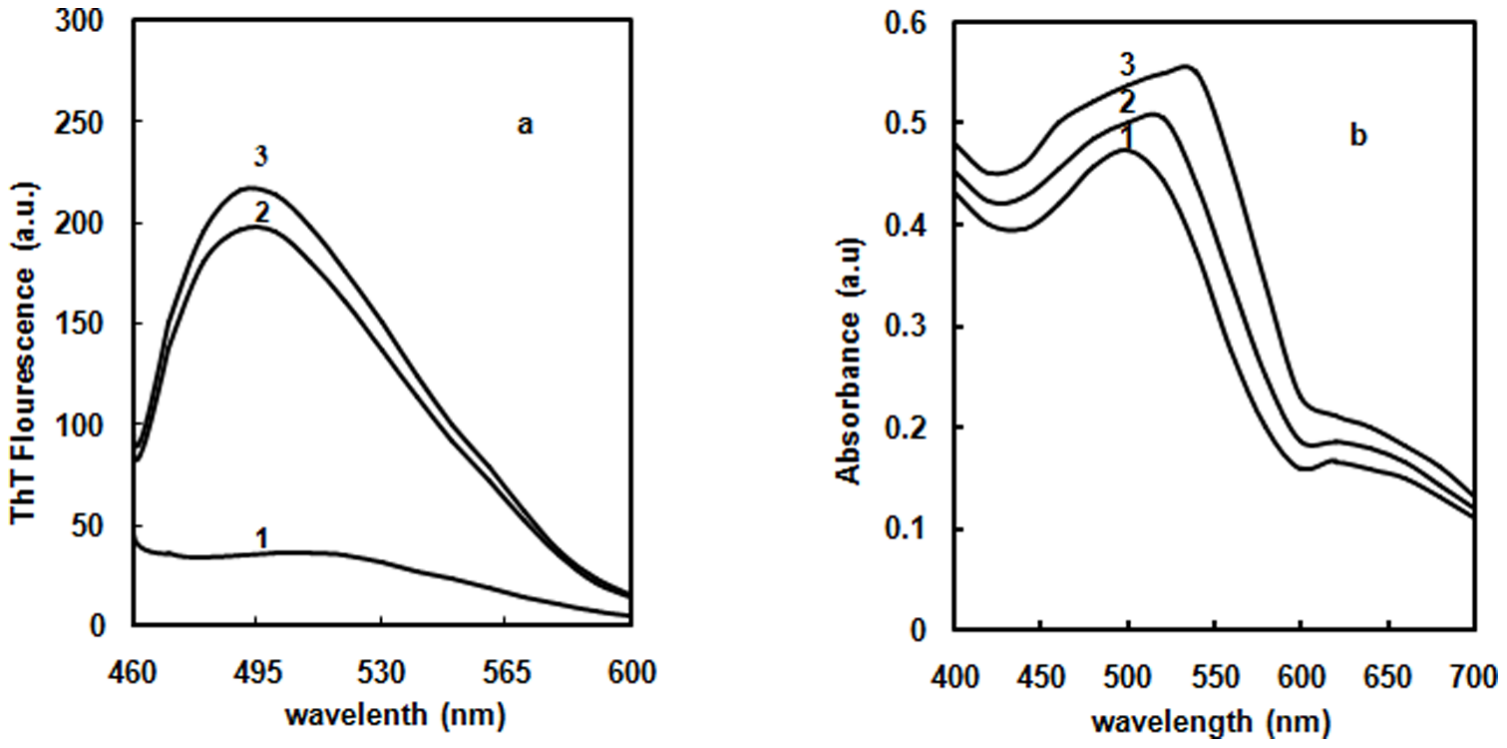
ThT and CR binding. (**a**) ThT emission spectra of native Hb in absence of glyoxal (curve 1); in the presence of 70% glyoxal (curve 2) and 30% glyoxal on day 20 (curve 3). The path length was 1 cm, λ_ex_ 440 nm and λ_em_ 460–600 nm. The protein concentration was 3 µM. (**b**) CR absorption spectra of native Hb in 20 mM sodium phosphate buffer (curve 1); in the presence of 70% glyoxal (curve 2) and 30% glyoxal on day 20 (curve 3). The protein concentration was 6 µM. The absorption spectra were recorded from 400–600 nm.

The intermolecular β-sheets observed in the presence of different concentrations of glyoxal were further assessed by using ThT, a benzothiazole dye that specifically binds to β-sheet aggregates. Glyoxal alone incubated with ThT was recorded and subtracted emission spectrum was represented here. After adding ThT, Hb was incubated with and without 70% glyoxal for 24 hrs. Native Hb did not bind to the dye hence exhibiting very low fluorescence intensity at 520 nm (curve 1 of Fig. 9a). A prominent 6 times increase in the fluorescence emission of this dye is observed at 490 nm upon binding of Hb in 70% glyoxal incubated protein relative to native (curve 2). Similarly, ThT is also added to Hb incubated with 30% glyoxal on day 20. There was slight increase in fluorescence intensity on day 20 (curve 3) relative to Hb in 70% glyoxal (curve 2). This suggests that AGEs have much greater amount of intermolecular β-sheets relative to aggregates. The results depicted that high β-sheet content of protein leads to the formation of aggregates as well as AGEs as seen by enhanced ThT fluorescence upon incubation at different time periods [36]. This also implies that 70% glyoxal induces an environment which enhances the formation of aggregates in native Hb whereas 30% glyoxal promotes the formation of AGEs on day 20.

### CR assay

To monitor the formation of aggregates, CR dye is diagnostically used. CR is a symmetrical sulfonated azo dye with a hydrophobic centre consisting of a biphenyl group spaced between the negatively charged sulfate groups. Amyloid binding to CR causes a red shift in the absorbance spectrum of the dye. CR or native Hb (data not shown) did not show any optical activity in the 400–700 nm spectral regions. Native Hb was mixed with CR and spectrum was acquired immediately. The spectrum showed a maximum peak around 500 nm, indicative of CR binding to Hb with distorted, asymmetric conformations (curve 1 of Fig. 9b) [37]. Glyoxal alone incubated with CR was monitored and here subtracted emission spectrum is reported. The CR spectra of Hb at 70%, and 30% glyoxal on day 20 red shifted the maximum peak around 520 nm (curve 2) and 540 nm (curve 3) respectively. This suggests that AGEs formed on day 20 at 30% glyoxal possess more intermolecular β-sheets in contrast to aggregates observed at 70% glyoxal. These results are consistent with the data obtained from ThT assay. It also means that increase in absorption is due to the formation of CR binding moieties which are formed upon incubating Hb with 70% glyoxal, and 30% glyoxal on day 20.

### Comet assay of Hb aggregates

**Figure 10:**
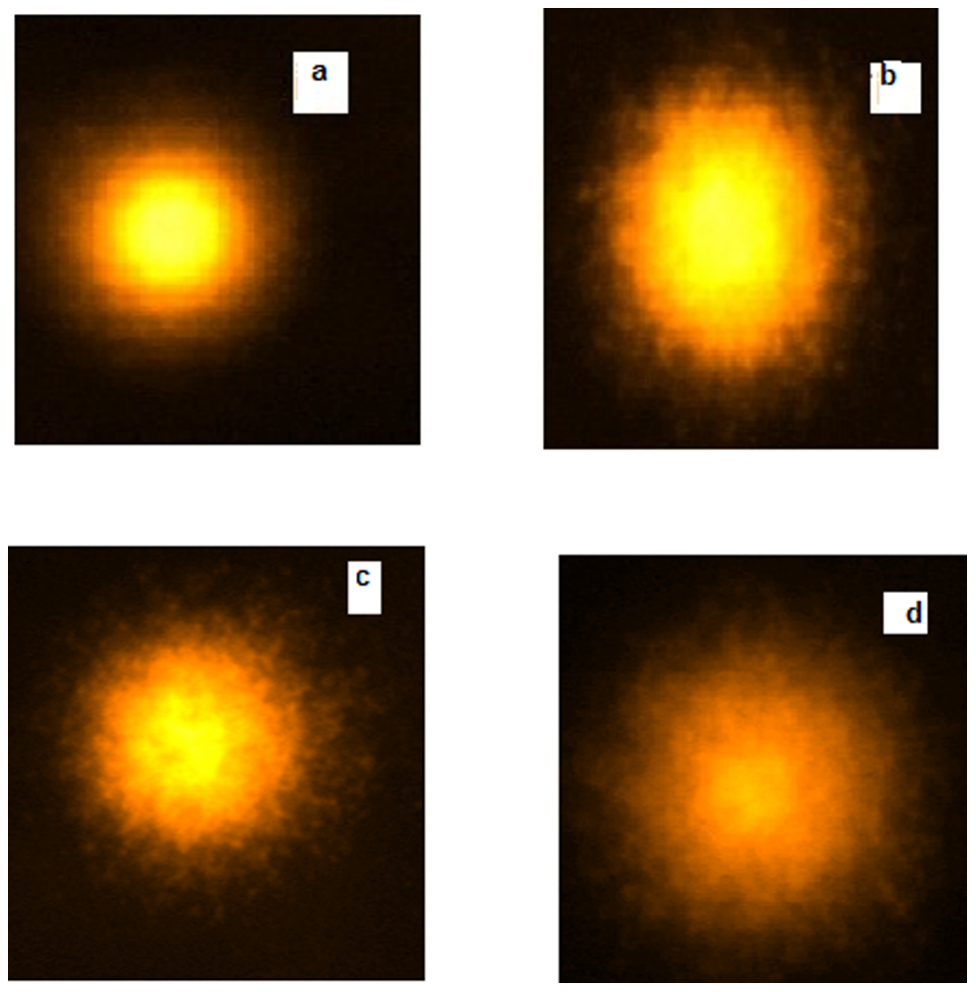
Comet assay. Images of treated lymphocyte nuclei (**a**) negative control; (**b**) in the presence of 3 µl of methyl methane sulfonate (25 µg/ml) as positive control (**c**) Hb aggregates formed at 70% glyoxal (**d**) glycated Hb at 30% glyoxal on day 20. The protein concentration was 50 µg.

To assess the genotoxic nature of Hb aggregates and AGEs, comet assay test was performed. This test detects nicks with high sensitivity in damaged single or double stranded DNA in very less amount. The lymphocytes were treated with aggregates of Hb in 70% glyoxal and AGEs in 30% glyoxal at day 20. The image of a negative control with a tail length of 5 µm showed that no effect was provided on lymphocytes in presence of 20 mM phosphate buffer of pH 7 (Fig. 10a) and the image of a positive control having a tail length of 17 nm depicted that lymphocytes were treated with methyl methane sulphonate (Fig. 10b). Hb at 70% glyoxal showed DNA damage of around 13 µm in tail length (Fig. 10c) whereas Hb incubated with 30% glyoxal on day 20 showed 18 µm in tail length (Fig. 10d). These results suggest that genotoxic effect is predominantly observed in glycated Hb relative to aggregated Hb in vitro. These aggregates and AGEs being small in size might get penetrated into the nuclear pore complex and caused the DNA damage [38].

### SEM studies

**Figure 11:**
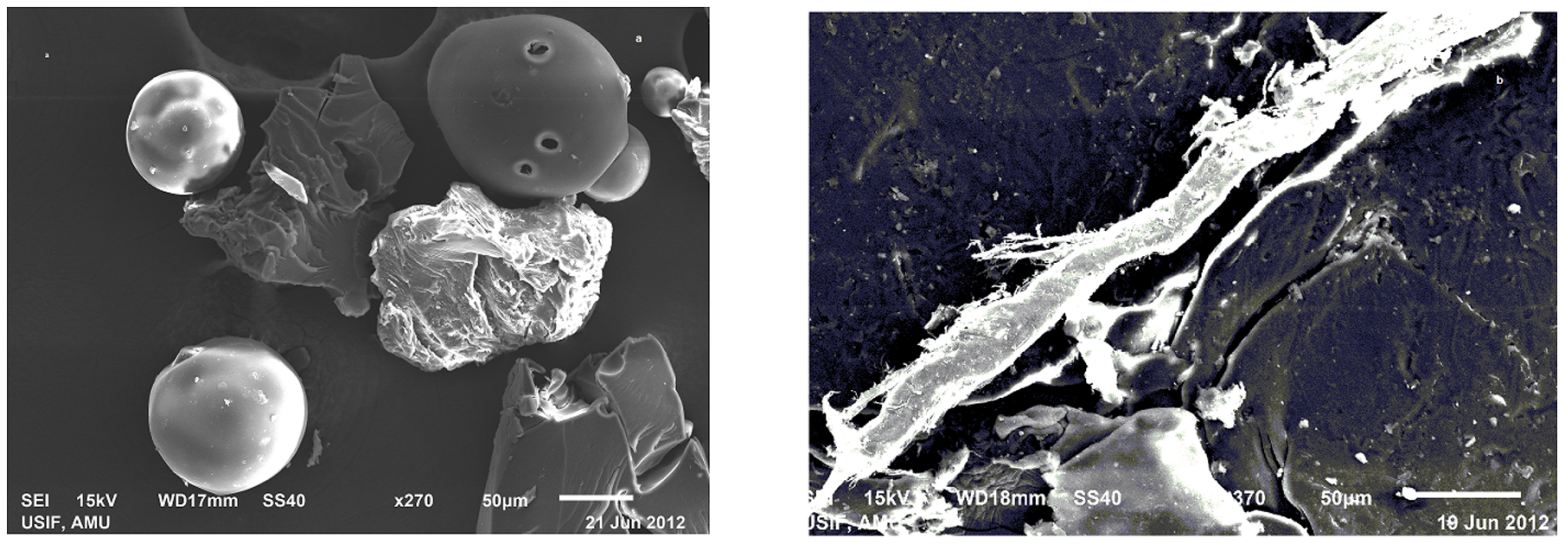
SEM images. (**a**) Hb aggregates at 70% glyoxal (**b**) AGE–Hb incubated at 30% glyoxal on day 20. The protein concentration was 150 µM.

To evaluate the structure and type of amyloids formed as a result of aggregation and glycation, Hb aggregates formed in presence of glyoxal were observed by SEM. Hb in presence of 70% glyoxal showed amorphous aggregates (Fig. 11a). SEM images of prolonged glycation of Hb at 30% glyoxal on day 20 revealed the presence of branched fibrils like aggregates (Fig. 11b). These results suggest that Hb forms amorphous aggregates at 70% glyoxal on incubating for 4 hrs and branch fibrils at 30% glyoxal on day 20 of incubation period.

## Discussion

Due to microbial activity and non-enzymatic autoxidation of oil or browning reactions of saccharides, glyoxal is commonly found in fermented food and beverages such as different varieties of beer, wine, and other beverages like black tea and sherry wine. It is also noticed in several fermented products such as soybean paste and yoghurt, bakery products like bread and edible oils. The general population is exposed mainly via consumption of glyoxal-containing food, but could be exposed through polluted air in urban areas and through traces of glyoxal in drinking-water. After oral exposure to glyoxal, major target organs such as pancreas and kidney are mainly affected. The toxic action of glyoxal leads to severe degenerative changes resembling those induced during diabetes. In animal studies, 30% and 40% aqueous glyoxal cause slight to definite skin irritations, depending on the application time. Glyoxal is directly genotoxic to bacterial and mammalian cells in vitro environment. The concentration of glyoxal in human blood plasma has been reported to be 0.1–1 µmol/litre, with higher levels reported for patients with diabetes or renal failure. In biological materials, less than 10% of glyoxal present is in unbound forms in aqueous solution (free glyoxal and hydrates), as most of the reactive carbonyl groups are reversibly bound to cysteinyl, lysyl, and arginyl residues of proteins that ultimately led to the formation of advanced glycation end-product (AGEs).

Glycated Hb is minor component of human Hb that are formed non-enzymatically by condensation of α or β chains of Hb. The subfraction HbA_1c_, a non enzymatic glycation at the amino-terminal valine residue of the β chain is found to be an important biomolecular marker for evaluating long term control of diabetes. Therefore study on glyoxal can be the effective approach to cure aggregation and glycation related problems. The significance of using glyoxal in our study is that due to the presence of dicarbonyl groups, it results in faster glycation reaction than glucose. Therefore, incubating Hb with glyoxal for longer time periods is the prevalent way to induce more formation of AGEs over glucose.

These results taken together, i.e. partially exposed Trp residues, retention of Soret band, exposure of hydrophobic clusters as indicated by increase ANS binding, considerable retention of secondary structure and loss of tertiary structure provide strong evidence that Hb forms MG state at 20% glyoxal. On further increasing organic solvent concentration, aggregation of Hb was induced at 70% as confirmed by exposed heme [39], less exposed hydrophobic surface, increase β-sheet secondary structure, disrupted tertiary structure, and extension of hydrodynamic size. Such observed enlargement of hydrodynamic size can be explained in terms of higher proportion of β sheet structure, a precursor of protein aggregation. As can be shown from ANS fluorescence, ThT and CR assays, preferential binding of glyoxal to partially unfolded protein molecules shifts the equilibrium from native states towards aggregate state. Comet assay proved the nature of aggregates to be genotoxic in nature and SEM studies confirmed the amorphous structure of Hb aggregates. These findings suggested that as MG state is deduced at 20% gloxal beyond it protein started to form aggregates [40]–[43]. Because the partially unfolded molecules are aggregation-competent, glyoxal binding greatly accelerates protein aggregation.

The most probable mechanism by which glyoxal induces formation of a compact destabilized protein conformation is that glyoxal, being the small molecule penetrates the protein structure and forms electrostatic and hydrogen bonds between carbonyl groups of glyoxal and amino acid residues of Hb. As the concentration of glyoxal increases, the polarity of the solvent decreases causing the disruption of native conformation of protein, hence resulting in exposure of tryptophan and heme to the solvent. Thus, glyoxal perturb the structure of protein surface, partially modifying the layer of water and native tertiary structure.

Hb concentration used in glycation study is 8 µM well below that which occurs in red blood cells (RBCs). According to law of mass action, rate of reaction depends on all of the reactants present in a reaction. Therefore, given the rates are equal, in vitro, the damage will be higher if the concentration of protein and glycating agent (glyoxal) is low and high respectively. Any mutation that shifts the equilibrium towards aggregates is thus likely to enhance the glycation induced damage. Therefore we performed glycation studies in Hb at 30% and 40% glyoxal concentrations. 30% glyoxal concentration was found to be more effective in forming cross-linked species as in 40% glyoxal, protein was aggregating. The glycation reaction was observed in Hb incubated with 30% glyoxal for 20 days at 37°C. This study resulted in the formation of new fluorophore adducts that can fluoresce with a λ_max_ of 450 nm upon excitation at 280 nm. The overlapping of emission peaks of Trp and glycation product around 375 nm indicates existence of an energy transfer process between Trp and AGE product which has excitation maxima near the emission maxima of Trp. This directs quenching of Trp fluorescence at the expense of AGEs fluorescence. The peak at 450 nm points towards the development of AGEs and the increase in its intensity suggests their accumulation. It has been confirmed that Hb forms AGEs on day 20 of incubation, as confirmed by λ_max_ at 330 and 365 nm [44]. Hb forms two different types of AGEs during progression of glycation. The progress of the reaction on the other hand also induces a conformational switch over in Hb, which destroys the heme pocket as well as weakens the heme-globin bond. The Soret peak initially shifted towards the met-Hb form (peak at 405 nm). Met-Hb formation further assists alterations in heme from Hb during glycation. The distorted heme associated with glycation of Hb may be imperative in dealing with diabetic problems co-occurring with anaemia. Moreover, glycation of protein with glyoxal leads to α-helix to β-sheet transition as confirmed by far-UV CD and FTIR spectra. Hb-AGE is characterized by loss in tertiary structure as depicted by near UV CD spectra. In addition to this, DLS results confirmed the presence of crosslinked structures in AGEs which were further confirmed by ThT and CR assays. Both these dyes bind to enhanced β-sheet conformations. SEM results confirmed that AGEs possess branched fibrils like aggregates.

**Figure 12:**
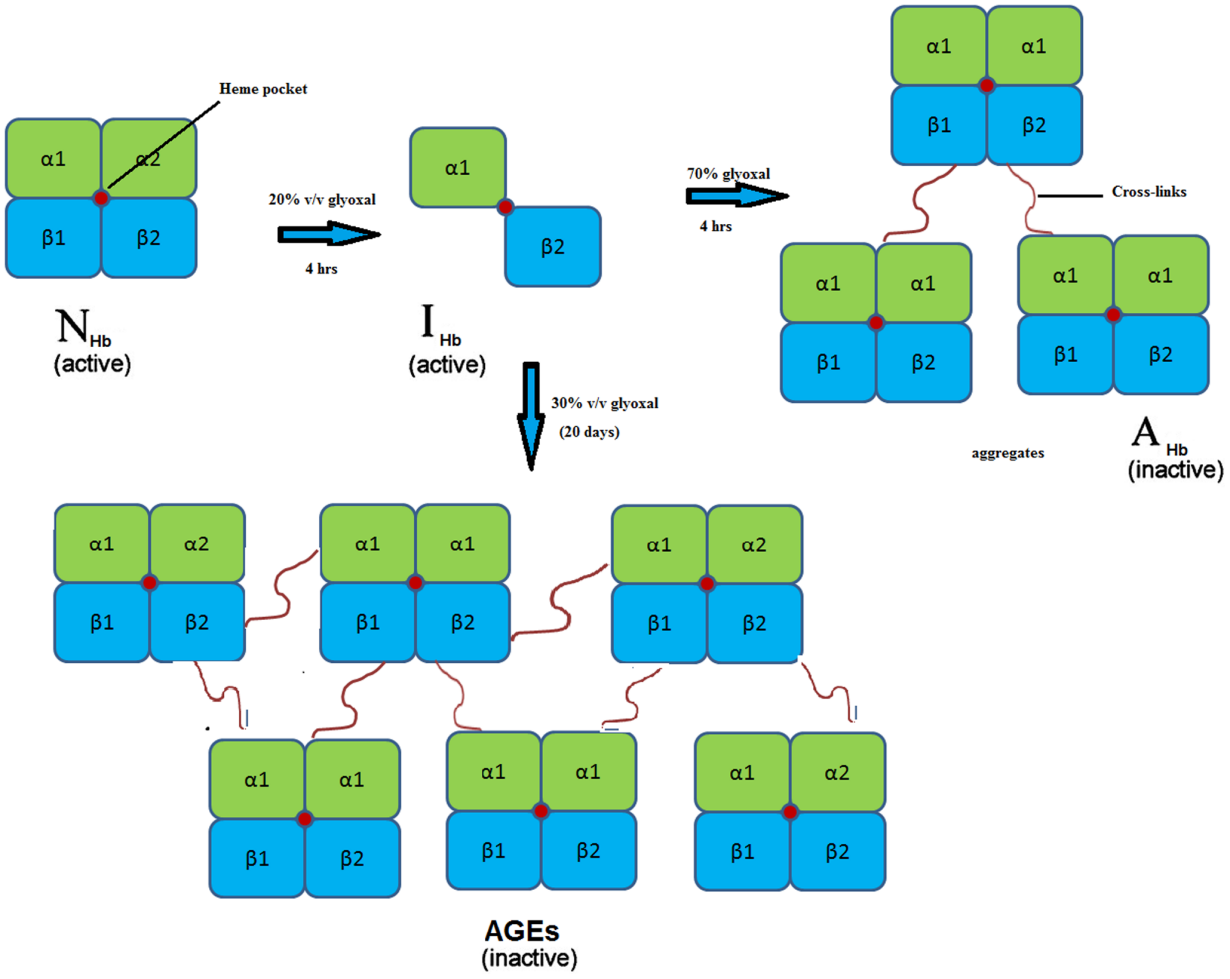
Scheme. Diagrammatic representation of Hb aggregation and glycation pathway.

A diagrammatic representation of the complete pathway was depicted Figure 12.

## Conclusion

The biological importance of non-native protein conformations ranging from various partially unfolded conformations to aggregated forms is continuously emphasized by recent research. These studies will be useful for revealing the origin of amyloid fibril formation in disease conditions. It would be a promising approach to cure amyloidogenic diseases by discovering new strategies for disaggregation to occur. Moreover, a better approach towards the pharmacodynamics of protein glycation occurred by sugar derivatives is obligatory in order to make AGEs as therapeutic target of pathologies. This is because diabetic hyperglycaemia habitually directs increase non-enzymatic glycation of Hb molecules resulting in more AGEs. Hence AGEs are regarded as the most trustworthy and promising indicator of diabetic control. Humans consuming glyoxal in form of fermented food or beverages are likely to have more glycation induced protein damage (or AGEs formation). The emergence of β structure with glycation also suggests possible therapeutic approaches based on negative regulation of β structure formation. According to this scenario, a putative glycation inhibitor might function by arresting the alpha structure or minimizing the β-structure formation.
